# Possible utilization of salivary IFN‐γ/IL‐4 ratio as a marker of chronic stress in healthy individuals

**DOI:** 10.1002/npr2.12157

**Published:** 2021-01-19

**Authors:** Yuika Takemori, Daimei Sasayama, Yukiyo Toida, Minori Kotagiri, Nobuhiro Sugiyama, Masaki Yamaguchi, Shinsuke Washizuka, Hideo Honda

**Affiliations:** ^1^ Department of Health Sciences Shinshu University Graduate School of Medicine Matsumoto Japan; ^2^ Mental Health Clinic for Children Shinshu University Hospital Matsumoto Japan; ^3^ Department of Psychiatry Shinshu University School of Medicine Matsumoto, Nagano Japan; ^4^ Department of Child and Adolescent Developmental Psychiatry Shinshu University School of Medicine Matsumoto Japan; ^5^ Department of Applied Occupational Therapy Shinshu University School of Health Sciences Matsumoto Japan; ^6^ Department of Mechanical Engineering & Robotics Shinshu University Graduate School of Science & Technology Ueda Japan

**Keywords:** attention, cytokines, psychological stress, saliva, sleep

## Abstract

**Introduction:**

Several studies show that psychological stress reduces Th1/Th2 ratio in blood samples. However, evidence is scarce regarding the cytokine alterations during stress in saliva. We investigated the influence of chronic stress on Th1/Th2 ratio and cytokine profiles in the saliva of healthy individuals. Further, we examined the associations of the salivary cytokine levels with sleep and attention problems, which are closely related with psychological stress.

**Methods:**

Salivary levels of 27 cytokines were measured by multiplex bead array assays in 31 healthy young individuals (health science students and hospital staff consisting of 11 men and 20 women, mean age [standard deviation] =21.5 [0.8] years). The Kessler Psychological Distress scale (K10) and Athens Insomnia Scale (AIS) were administered to assess subjective chronic psychological stress and sleep problems. Further, participants were asked to wear Actigraph GT3X accelerometers for 3 days to assess the total sleep time. Attention problems were assessed by administering the Integrated Visual and Auditory Continuous Performance Test (IVA‐CPT).

**Results:**

Thirteen cytokines with >80% detectable results were included in the main analyses. The IFN‐γ/IL‐4 ratio, which is a commonly used index for Th1/Th2 ratio, showed significant negative correlations with the K10 and AIS scores. None of the cytokines were significantly associated with sex, body mass index, sleep index measured by Actigraph, or IVA‐CPT scores.

**Conclusion:**

Chronic stress may be associated with alterations of the Th1/Th2 balance in salivary cytokine production. IFN‐γ/IL‐4 ratio in saliva may serve as a potential biomarker of chronic stress in healthy individuals.

## Abstract 2

The present study investigated the influence of chronic stress on Th1/Th2 ratio and cytokine profiles in the saliva of healthy individuals. The IFN‐γ/IL‐4 ratio showed a significant negative correlation with chronic stress, assessed by the Kessler Psychological Distress scale. IFN‐γ/IL‐4 ratio in saliva may serve as a potential biomarker of chronic stress in healthy individuals.

## Graphical abstract

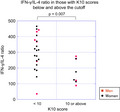

## 1 INTRODUCTION

Cytokine alterations in the blood and cerebrospinal fluid of patients with psychiatric diseases have consistently been reported in the literature.^1^, ^2^, ^3^, ^4^ Alterations in the cytokine network in psychiatric patients could be related to stress induced by the disease as well as to the pathophysiology of the disease. Indeed, the common immune alterations observed in chronically ill psychiatric patients are also observed in healthy individuals with chronic stress.^5^, ^6^

Stress in humans is known to down‐regulate Th1 cytokines and up‐regulate Th2 cytokines.^7^ An imbalance between Th1 and Th2 cytokines during psychological stress has been reported in various studies. For example, studies have consistently reported that academic stress results in decreased Th1/Th2 ratio in blood samples^8^, ^9^ and in stimulated peripheral blood mononuclear cells.^9^, ^10^

Other known alterations of cytokines associated with stress has been summarized in a meta‐analysis by Marsland et al.^11^ Their findings demonstrated stress‐related increases of circulating interleukin (IL)‐6, IL‐1β, IL‐2, IL‐10, and tumor necrosis factor‐α (TNF‐α). Their meta‐analysis on two studies also showed a significant stress‐related increase of circulating IL‐4 levels. In contrast, existing evidence did not support the increase in the circulating levels of IL‐1 receptor antagonist or interferon (IFN)‐γ in response to stress. Their finding, which demonstrated a stress‐related increase in IL‐4 levels without a significant change in IFN‐γ levels, also suggests that the Th1/Th2 balance, which is often represented by the IFN‐γ/IL‐4 ratio, may be decreased due to stress.

A few studies have shown that some cytokines are altered in saliva in response to psychological stress, suggesting the potential utility of salivary cytokines as markers of stress. Particularly, previous studies reported that acute stress in healthy individuals increased salivary levels of IL‐1β,^12^, ^13^ TNF‐α, and IL‐6.^14^, ^15^ Lester et al^16^ showed that chronically elevated salivary levels of IL‐2, IL‐6, and IL‐12 were also observed after academic stress. Furthermore, Martínez et al^17^ showed that fear and chronic stress were related to elevated levels of proinflammatory cytokines in healthy individuals. A study in mice also showed chronic stress‐induced elevation of IL‐1β in saliva.^18^

Sleep disruption, which is bidirectionally associated with psychological stress, is also known to influence cytokine levels. Previous studies showed that sleep deprivation increased IL‐6 and TNF in plasma^19^, ^20^ and IL‐1β, IL‐6, and IL‐17 in stimulated cells.^21^ Contrarily, a recent study showed that children with longer and more efficient sleep had higher salivary levels of IL‐1β and IL‐6 upon awakening,^22^ complicating the interpretation of how sleep influences the immune system. Another study showed that children with obstructive sleep apnea had higher circulating levels of IL‐17 and IL‐23 and lower scores in neurocognitive tests^23^; furthermore, low scores in cognitive tests, such as decreased alertness and increased inattention, were significantly related with the abnormal levels of these cytokines. Other studies have also shown the associations of sleep and attention problems with cytokine levels.^24^, ^25^ Thus, accumulating evidence suggests that disrupted sleep and disturbed attention, along with psychological stress, are important factors associated with cytokine alterations.

The main aim of the study, therefore, was to investigate the influence of chronic stress on cytokine profiles and Th1/Th2 ratio in the saliva of healthy individuals. We further aimed to evaluate sleep and attention problems in participants to elucidate the relationships between stress, sleep, attention, and the cytokine alterations in saliva.

## 2 METHODS

### 2.1 Participants

Participants were students of Shinshu University School of Health Sciences and staff of Shinshu University Hospital (11 men and 20 women, mean age [standard deviation] =21.5 [0.8] years). None of the hospital staff or students participating in the study were engaged in night shift work. Students participating in the study were third‐ or fourth‐year students. Sample collection from students was not performed within a month after the examination or during the pre‐examination period. Therefore, the students were likely to be free from excessive academic stress 30 days prior to participation of the study. All subjects were biologically unrelated Japanese individuals. Participants were excluded if they reported a prior medical history of central nervous system disease, psychiatric disorder, or if they suffered from any inflammatory, infectious, or systemic immune diseases or periodontal disease at the time of inclusion in the study. The study protocol was approved by the ethics committee at the Shinshu University School of Medicine, Japan. Written informed consent was obtained from all participants after description of the study.

### 2.2 Saliva collection

Saliva samples were collected at about 10:00 am (9:40‐10:50 am). Drinking, eating, or brushing teeth were not allowed 30 minutes before saliva collection. Approximately 150 μL of whole saliva was collected by passive‐drool method. None of the saliva samples were visibly contaminated with blood. Saliva samples were stored at −80℃ and thawed at 4℃ in a refrigerator just prior to analysis.

### 2.3 Cytokine measurement

Cytokine levels in saliva were measured by a multiplex bead immunoassay assay system (Cat. #M500KCAF0Y, Bio‐Plex Pro Human Cytokine Grp I Panel 27‐Plex; Bio‐Rad Laboratories, Inc, Tokyo, Japan) as described previously.^26^, ^27^ Previous studies have shown that Bio‐Plex multiplex bead array immunoassay system, which was originally developed by Luminex Co., is a reliable method of measuring human cytokine levels.^28^, ^29^ The following 27 cytokines were analyzed according to the manufacturer's instructions: fibroblast growth factor (FGF) basic, eotaxin, granulocyte colony‐stimulating factor (G‐CSF), granulocyte‐macrophage colony‐stimulating factor (GM‐CSF), IFN‐γ, IL‐1β, IL‐1 receptor antagonist, IL‐2, IL‐4, IL‐5, IL‐6, IL‐7, IL‐8, IL‐9, IL‐10, IL‐12p70, IL‐13, IL‐15, IL‐17, IFN‐γ‐induced protein‐10 (IP‐10), monocyte chemotactic protein (MCP)‐1, macrophage inflammatory protein (MIP)‐1α and (MIP)‐1β, platelet‐derived growth factor (PDGF)‐BB, regulated on activation normal T‐cell expressed and secreted (RANTES), TNF‐α, and vascular endothelial growth factor (VEGF). The plates were read on a Bio‐Plex Array Reader (Bio‐Plex 200 System and Bio‐Plex Manager Version 6.1, Bio‐Rad Laboratories, Inc, Tokyo, Japan). All samples were measured in duplicate in a single assay run. Cytokines with >80% detectable results were included in our main analyses. We also performed additional analyses after assigning 0 pg/mL to concentrations under the detection limit. The IFN‐γ/IL‐4 ratio was used as the index for Th1/Th2 balance of cytokines in saliva, as is commonly used in previous studies.^30^, ^31^

### 2.4 The Kessler psychological distress (K10) scale

Psychological stress was assessed using the K10 scale, a self‐reported measure of psychological stress that includes 10 questions about one's emotional state within a 30‐day reference period.^32^ The participants were asked to answer each of the 10 questions based on a scale of 0 (none of the time) to 4 (all of the time). A cutoff point of ≥10 has been proposed as an optimal score for screening mood and anxiety disorders in Japanese populations.^33^

### 2.5 Athens insomnia scale (AIS)

Sleeping problems were assessed using the Japanese version of the AIS, a commonly used self‐rating inventory consisting of eight items to assess the severity of insomnia. The Japanese version of the AIS has been shown to have sufficient validity and diagnostic utility.^34^

### 2.6 The integrated visual and auditory continuous performance test (IVA‐CPT)

Attention and response inhibition of the participants were measured by the IVA‐CPT (BrainTrain, Inc, Richmond, VA).^35^ The IVA‐CPT yields standardized scores of attention and response control. The full‐scale attention quotient and response control quotient were used as the index of attention.

### 2.7 Total sleep time

The average total sleep time for 3 nights was measured using the triaxial accelerometer (ActiGraph wGT3X‐BT, ActiGraph Corp, Pensacola, FL). Participants wore the accelerometer for 3 days prior to other assessment measures and saliva collection.

Duration of sleep was calculated as the time from sleep onset time to final sleep offset time. Total sleep time was calculated as duration of sleep minus wake after sleep onset (WASO). Sleep onset was defined as the first minute that was followed by a 10‐minute immobility period that contained no more than one epoch with any motion count. Sleep offset was defined as the last minute following a 10 consecutive Sadeh + inclinometer algorithm (SIA)‐scored sleep minutes ^36^. Sleep onset time was defined as the time of first sleep onset after the going to bed time if the epoch at the time of going to bed is a SIA‐scored wake minute, or the time of first sleep onset after the closest 5 consecutive SIA‐scored wake minutes before the going to bed time if the epoch at the time of going to bed is a SIA‐scored sleep minute. Sleep offset time was defined as the time of first sleep offset after the sleep onset time with at least 30 minutes of SIA‐scored wake minutes before the next 10 consecutive SIA‐scored sleep minutes. WASO was defined as the number of SIA‐scored wake minutes between sleep onset time to sleep offset time.

### 2.8 Statistical analysis

Differences in continuous variables were assessed by the Mann‐Whitney *U*‐test or unpaired Student's *t*‐test, depending on the data distribution. Associations between categorical variables were assessed by Fisher's exact test. Associations of salivary cytokine levels with clinical variables were assessed using the Spearman's correlation coefficients. A value of *P* < .05 was considered statistically significant. All analyses were performed using the statistical package for the social sciences (SPSS) version 26 (IBM Corp, Armonk, NY) and R software version 3.6.3.^37^

## 3 RESULTS

The demographic and clinical characteristics of the participants are summarized in Table 1. When men and women were compared, no significant difference was found except for body height and body weight.

**TABLE 1:** Demographics and clinical characteristics

	Women	Men	Statistics
n	20	11	
Age (y)	21.3 (0.7)	21.9 (0.9)	*t* = 1.90, *df* = 15.46, *P* = .08
Height (cm)	158.8 (4.9)	173.2 (9.6)	*t* = 1.65, *df* = 12.91, *P* < .001
Body Weight (kg)	52.1 (7.1)	67.1 (14.9)	*t* = 3.13, *df* = 12.54, *P* = .008
BMI	20.6 (2.1)	22.2 (3.8)	*t* = 1.32, *df* = 13.60, *P* = .21
K10			
K10 score	5.3 (4.4)	8.1 (6.7)	*t* = 1.27, *df* = 14.81, *P* = .23
Above cutoff (10 or higher)	4	5	*P* = .14
IVA‐CPT			
Attention quotient	118.6 (11.7)	103.8 (15.5)	*t* = −0.82, *df* = 19.37, *P* = .42
Response control quotient	109.3 (13.8)	106.6 (13.4)	*t* = −0.16, *df* = 18.39, *P* = .87
AIS score^a^	2.9 (1.4)	2.5 (1.6)	*t* = 1.23, *df* = 12.69, *P* = .24
Duration of sleep (min)^b^	387.0 (108.5)	328.9 (82.6)	*t* = −1.72, *df* = 23.4, *P* = .099
Total sleep time (min)^b^	340.5 (96.1)	284.5 (72.0)	*t* = −0.84, *df* = 22.01, *P* = .41

Note

Data are shown as mean (standard deviation).

Abbreviations: AIS, Athens Insomnia Scale; BMI, body mass index; IVA‐CPT, Integrated Visual and Auditory Continuous Performance Test; K10, Kessler Psychological Distress Scale 10.

^a^ One missing value in women.

^b^ One missing value in men.

**FIGURE 1:**
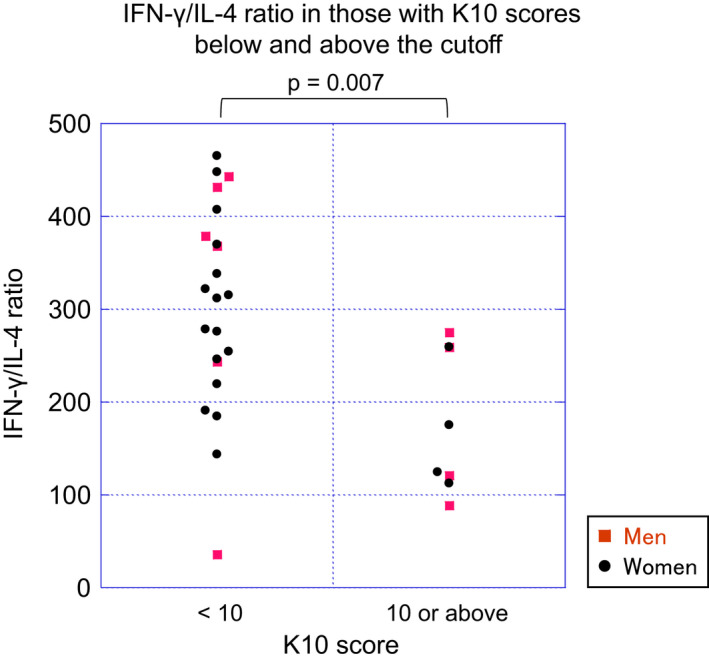
IFN‐γ/IL‐4 ratio in those with K10 scores below and above the cutoff. IFN‐γ/IL‐4 ratios in those with K10 scores below and above the cutoff of 9/10 are shown. The IFN‐γ/IL‐4 ratio was significantly lower in those with K10 above the cutoff (*P* = .007, Mann‐Whitney's test). Each of the 10 questions of the K10 was scored on a scale of 0 (none of the time) to 4 (all of the time)

**FIGURE 2:**
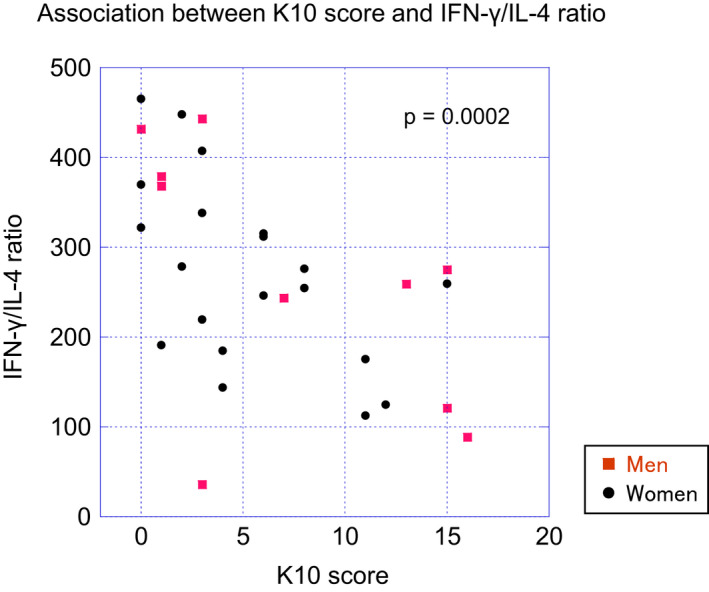
Association between K10 scores and IFN‐γ/IL‐4 ratio. A significantly negative correlation was found between the IFN‐γ/IL‐4 ratio and K10 score (*ρ* = −0.63, *P* = .0002). Each of the 10 questions of the K10 was scored on a scale of 0 (none of the time) to 4 (all of the time)

Table 2 shows the detection rate and the mean and range of the concentration of the cytokines as well as the sensitivity and the precision of the assay system. Figure S1 shows the intercorrelations between cytokines. None of the cytokines were significantly correlated with sex, BMI, IVA‐CPT scores, duration of sleep, or total sleep time. No significant difference in the levels of any of the cytokines with >80% detection rate was found between those with K10 scores above and below the cutoff. However, as shown in Figure 1, the IFN‐γ/IL‐4 ratio was significantly lower in those with K10 above the cutoff (*P* = .007). Figure 2 depicts the negative correlation observed between the IFN‐γ/IL‐4 ratio and K10 score (*ρ* = −0.63, *P* = .0002). The IFN‐γ/IL‐4 ratio was also significantly correlated with the AIS score (*ρ* = −0.47, *P* = .009) but not with other clinical parameters (Figure S2). The IFN‐γ/IL‐4 ratio and K10 score were significantly correlated even after controlling for the AIS score (*ρ* = −0.56, *P* = −0.002; partial Spearman rank test).

**TABLE 2:** Salivary cytokine concentrations

Cytokines	Detection rate	Mean (SD) (pg/mL)^a^	Range (pg/mL)^a^	Assay sensitivity (pg/mL)^b^	Intra‐assay %CV^b^	Inter‐assay %CV^b^
IL‐1β	100.0%	6.33 (9.12)	0.30‐40.65	0.24	3.6	3.2
IL‐1RA	96.8%	1614.70 (1295.17)	199.44‐4909.94	3.16	4.7	5.1
IL‐2	45.2%	4.54 (6.24)	1.43‐23.56	0.75	1.7	2.5
IL‐4	96.8%	0.95 (1.17)	0.28‐6.09	0.09	3.2	1.9
IL‐5	16.1%	17.67 (17.41)	3.34‐44.53	0.86	2.3	2.3
IL‐6	93.5%	3.60 (10.13)	0.42‐55.67	0.34	2.2	3.0
IL‐7	74.2%	54.83 (78.94)	5.70‐345.92	1.22	2.7	3.9
IL‐8	100.0%	180.06 (170.73)	17.59‐604.49	0.36	3.2	2.8
IL‐9	61.3%	14.911 (14.91)	2.61‐69.93	1.08	2.6	7.1
IL‐10	12.9%	12.21 (10.54)	2.12‐26.86	0.69	2.3	3.4
IL‐12p70	41.9%	6.60 (9.69)	1.56‐35.35	0.78	3.3	2.9
IL‐13	48.4%	2.23 (3.00)	0.16‐11.37	0.22	3.1	2.7
IL‐15	16.1%	108.44 (58.96)	49.69‐195.82	12.82	2.8	4.1
IL‐17	35.5%	7.23 (8.65)	2.24‐30.34	1.16	2.4	1.4
Eotaxin	87.1%	1.89 (2.60)	0.35‐12.72	0.05	4.4	1.2
FGF basic	100.0%	8.22 (4.72)	3.68‐28.03	2.54	3.1	2.4
G‐CSF	16.1%	85.46 (129.63)	12.69‐314.61	3.63	3.1	4.0
GM‐CSF	12.9%	5.13 (2.32)	2.46‐8.11	0.19	4.3	2.2
IFN‐γ	100.0%	175.58 (67.21)	68.83‐327.30	1.05	3.1	3.6
IP‐10	100.0%	913.06 (879.15)	82.39‐3631.05	1.43	2.8	6.0
MCP‐1	93.5%	52.14 (35.32)	5.55‐132.49	0.44	3.2	3.4
MIP‐1α	100.0%	0.82 (0.75)	0.13‐3.35	0.06	4.5	4.2
PDGF‐BB	51.6%	194.75 (320.58)	2.30‐1188.32	2.96	3.3	9.7
MIP‐1β	96.8%	4.08 (3.31)	0.38‐10.65	1.41	3.4	2.5
RANTES	74.2%	5.65 (8.14)	0.80‐33.89	3.98	3.0	6.7
TNF‐α	96.8%	22.75 (27.39)	2.02‐136.10	1.13	3.5	3.0
VEGF	54.8%	426.55 (452.01)	39.27‐1697.90	10.16	2.8	8.5
IFN‐γ/IL‐4	96.8%	269.64 (115.38)	35.69‐465.25	n/a	n/a	n/a

^a^ Assayed using high‐sensitivity photomultiplier tube (PMT) detector.

^b^ Data derived under standard PMT setting, provided by the manufacturer.

When the same analyses were performed after assigning 0 pg/mL to undetected values, IL‐7 and IL‐13 levels were significantly higher in those with K10 above the cutoff (both *P* = .046). The IL‐7 and IL‐13 levels showed significantly positive correlations with K10 scores (IL‐7: *ρ* = 0.38, *P* = .037; IL‐13: *ρ* = 0.37, *P* = .041). However, neither IL‐7 nor IL‐13 showed significant correlations with other parameters (sex, BMI, IVA‐CPT scores, duration of sleep, or total sleep time) or with the AIS score.

## 4 DISCUSSION

The present study showed that the IFN‐γ/IL‐4 ratio in saliva was negatively correlated with psychological stress assessed by K10. The finding is in line with previous studies showing down regulation of Th1 cytokines and up regulation of Th2 cytokines during examination stress in blood^8^, ^9^ and stimulated peripheral mononuclear cells.^9^, ^10^ However, the present study is the first, to our knowledge, to report the association of stress with Th1/Th2 balance in saliva. The IFN‐γ/IL‐4 ratio was not correlated with objective sleep index measured by actigraphy or with attention and response control measured by IVA‐CPT. Although the AIS score was significantly correlated with both the IFN‐γ/IL‐4 ratio and K10 score, the IFN‐γ/IL‐4 ratio and K10 score were significantly correlated even after controlling for AIS score. Therefore, it is unlikely that the correlation between IFN‐γ/IL‐4 and stress was spuriously caused by other stress‐related factors such as sleep or attention impairment.

Although the detection rates of IL‐7 and IL‐13 were low in our samples, these cytokines also showed significant associations with the K10 scores when the undetected levels were treated as 0 pg/mL. In line with these findings, previous studies also reported higher plasma IL‐7^38^ and serum IL‐13^39^ levels in patients with depression. However, due to the small number of detected levels of these cytokines in the present study, the associations of salivary IL‐7 and IL‐13 with psychological stress should be re‐examined in future studies.

Previous studies have reported that the mean and standard deviation values of the K10 and AIS scores in a healthy Japanese community sample were 6.1 (6.3)^33^ and 2.64 (2.02),^34^ respectively. These scores were comparable to those in the present study. IVA‐CPT scores reported in our study were also comparable to that previously reported in 45 university students in Japan (mean [standard deviation] attention quotient of 115.38 [14.14] and response control quotient of 106.89 [14.57]).^40^ Furthermore, the duration of sleep reported in the present study was similar to the previously reported average sleep duration of 6.1 hours in Japanese university students.^41^ Therefore, the participants in our study are likely to be representative of healthy university students in Japan. Thus, our findings may specifically apply to a young, healthy, and well‐educated population of individuals.

Studies reported increased salivary IFN‐γ/IL‐4 ratio in patients with oral lichen planus^31^ and primary Sjögren's syndrome.^30^ Such increase in Th1/Th2 balance indicates a shift away from humoral immunity (Th2) toward cellular immunity (Th1). In contrast, a stress‐induced immune response is known to shift the balance from Th1 to Th2 cytokine response.^10^ Both the cortisol and catecholamines released in blood in response to stress promote the shift from Th1 to Th2,^42^ leading to decreased Th1/Th2 balance in systemic cytokine production. However, the mechanism of inflammatory change in saliva during stress remains unclear.

Although low correlations between salivary and blood cytokines levels have been reported,^43^ a recent study showed that the change in the cytokine levels in plasma during stress was positively correlated with the change in the levels in saliva.^44^ Therefore, the salivary cytokine levels observed in the present study may have reflected the systemic immune shift from Th1 to Th2 induced by stress. Despite the necessity of further studies to elucidate the mechanism of salivary cytokine alterations during stress, our data add to the evidence that salivary cytokines may serve as a convenient tool to assess chronic stress.

One concern in using salivary cytokines as biomarkers is the scarce evidence regarding the stability of the salivary cytokine levels. However, one of the few studies that examined the short‐time reliability of salivary cytokine levels demonstrated a relatively high test‐retest correlation.^45^ In particular, the correlation between the 2 samples collected 2 hours apart from each of the 426 adolescent girls was generally high for the c‐reactive protein and 6 cytokines assessed (ie, IL‐1β, IL‐6, IL‐8, IL‐18, TNF‐α, and MCP‐1) (mean *r* = 0.67). Furthermore, their study also showed that the within‐person change between the 2 samples in each participant was negligible (|*d*| < 0.14). Thus, existing evidence suggests that salivary cytokines have enough stability to be used as biomarkers. However, IL‐4 and IFN‐γ, which were identified in our study as candidate markers for psychological stress, were not examined in the study by Shields et al,^45^ and therefore, further studies are needed to confirm the appropriateness of utilizing these cytokines as stable biomarkers.

Several previous studies have reported the presence of diurnal rhythm in salivary cytokine levels. Koizumi et al^27^ examined the salivary cytokine levels with the same cytokine panel used in the present study and reported significant diurnal variation for IL‐1 receptor antagonist, IL‐7, IL‐8, IL‐10, IL‐13, eotaxin, IFN‐γ, and TNF‐α. Their study reported that the salivary levels of most of the cytokines were highest in the morning. Similarly, other studies have also reported highest levels of IL‐1β^46^ and IL‐6^47^ in the morning. Although we did not assess the diurnal rhythm of the cytokines in the present study, all the samples were collected around 10:00 am to reduce the influence of diurnal variation. Therefore, the diurnal variation was unlikely to have caused any major bias in the results. However, further studies are needed to determine the best time of the day for collecting saliva when utilizing it as a stress marker.

There are several strengths in using salivary IFN‐γ/IL‐4 ratio for a biomarker of stress. First, both catecholamines and glucocorticoids, which are released in response to stress, may cause a selective suppression of Th1 functions and a shift toward a Th2 cytokine pattern.^48^ Considering the difference in the site of action between catecholamines and glucocorticoids, a biomarker which reflects the release of both chemical messengers will likely to be more sensitive to various responses to stress. Secondly, the ease and convenience of collecting saliva enables repeated collection of the samples in future studies. Therefore, future research could be designed to examine the temporal change and the diurnal patterns of the Th1/Th2 balance. Finally, non‐invasiveness of saliva collection makes this biomarker suitable for assessing stress in the clinical setting. However, as mentioned above, the stability and the reliability of salivary IFN‐γ/IL‐4 ratio has not been confirmed previously. Therefore, further studies are necessary before applying this biomarker in clinical practice.

Several limitations must be considered when interpreting our findings. The main limitation was the small sample size. Replication in larger studies is needed to confirm our findings. The second limitation was the cross‐sectional design of the study, which did not allow causal interpretations of the data. Another limitation was that the subjects were limited to health science students and hospital staff. Therefore, the results cannot be generalized to general population.

Despite these limitations, the present study showed for the first time the possibility of stress‐induced alteration of the Th1/Th2 balance of cytokine production in saliva. The finding suggests that IFN‐γ/IL‐4 ratio in saliva may serve as a potential biomarker of chronic stress in healthy individuals.

## CONFLICT OF INTEREST

None to declare.

## AUTHOR CONTRIBUTIONS

DS designed the study, and Y. Takemori and D.S wrote the draft of the manuscript. Y. Takemori, DS, Y. Toida, MK, and NS recruited participants and collected saliva samples. Y. Takemori and DS performed statistical analyses. NS, MY, HH, and SW supervised the data analysis and writing of the paper. All authors contributed to and have approved the final manuscript.

## APPROVAL OF THE RESEARCH PROTOCOL BY AN INSTITUTIONAL REVIEWER BOARD

The study was approved by the ethics committee at the Shinshu University School of Medicine, Japan.

## INFORMED CONSENT

Informed consent for study participation was obtained from every subject.

## Supporting information

Fig S1

Fig S2

## Data Availability

The data that support the findings of this study are available from the corresponding author upon reasonable request. The IRB did not grant the deposit of raw data in a publicly accessible data archive or repository at the time of approval since the procedure was not included in the study protocol or informed consent document.
