# Supplementary material for: Possible utilization of salivary IFN‐γ/IL‐4 ratio as a marker of chronic stress in healthy individuals
Source: Neuropsychopharmacol Rep. 2021 Jan 19;41(1):65–72. doi: 10.1002/npr2.12157 (PMC8182956; doi:10.1002/npr2.12157)
Supplement: Supplementary file 1 — Fig S1 [file NPR2-41-65-s001.pdf]

Figure S1: Intercorrelations between salivary cytokines

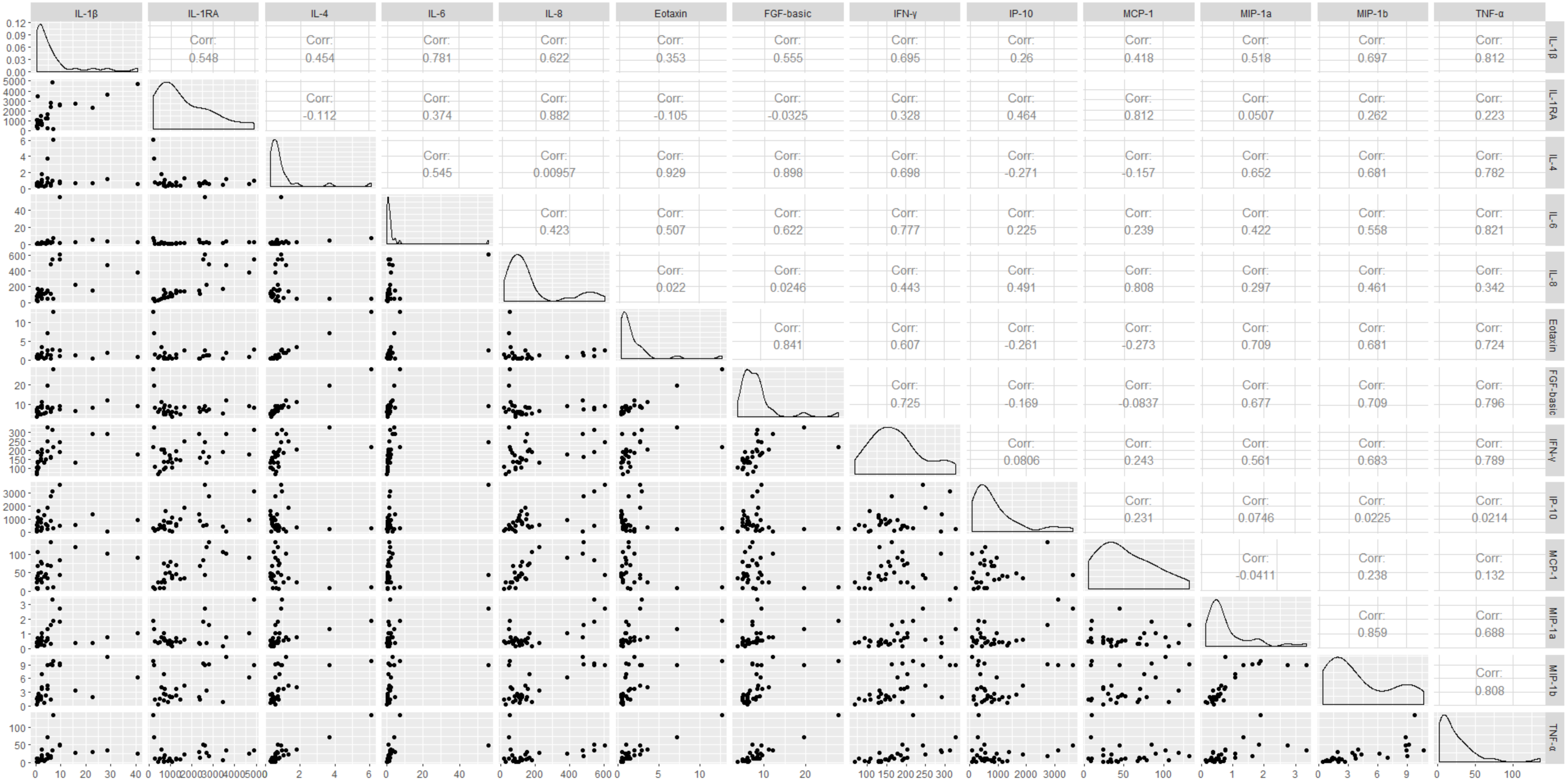

Figure S1: Intercorrelations between salivary cytokines. The distribution of each cyotkine is shown on the diagnal. Below the diagnal, the bivariate scatter plots are displayed. Above the diagnal, the values of the Spearman's correlation coefficients are shown.
