# Supplementary material for: Possible utilization of salivary IFN‐γ/IL‐4 ratio as a marker of chronic stress in healthy individuals
Source: Neuropsychopharmacol Rep. 2021 Jan 19;41(1):65–72. doi: 10.1002/npr2.12157 (PMC8182956; doi:10.1002/npr2.12157)
Supplement: Supplementary file 2 — Fig S2 [file NPR2-41-65-s002.pdf]

Figure S2: Spearman’s correlation coefficients between IFN-γ/IL-4 and clinical parameters

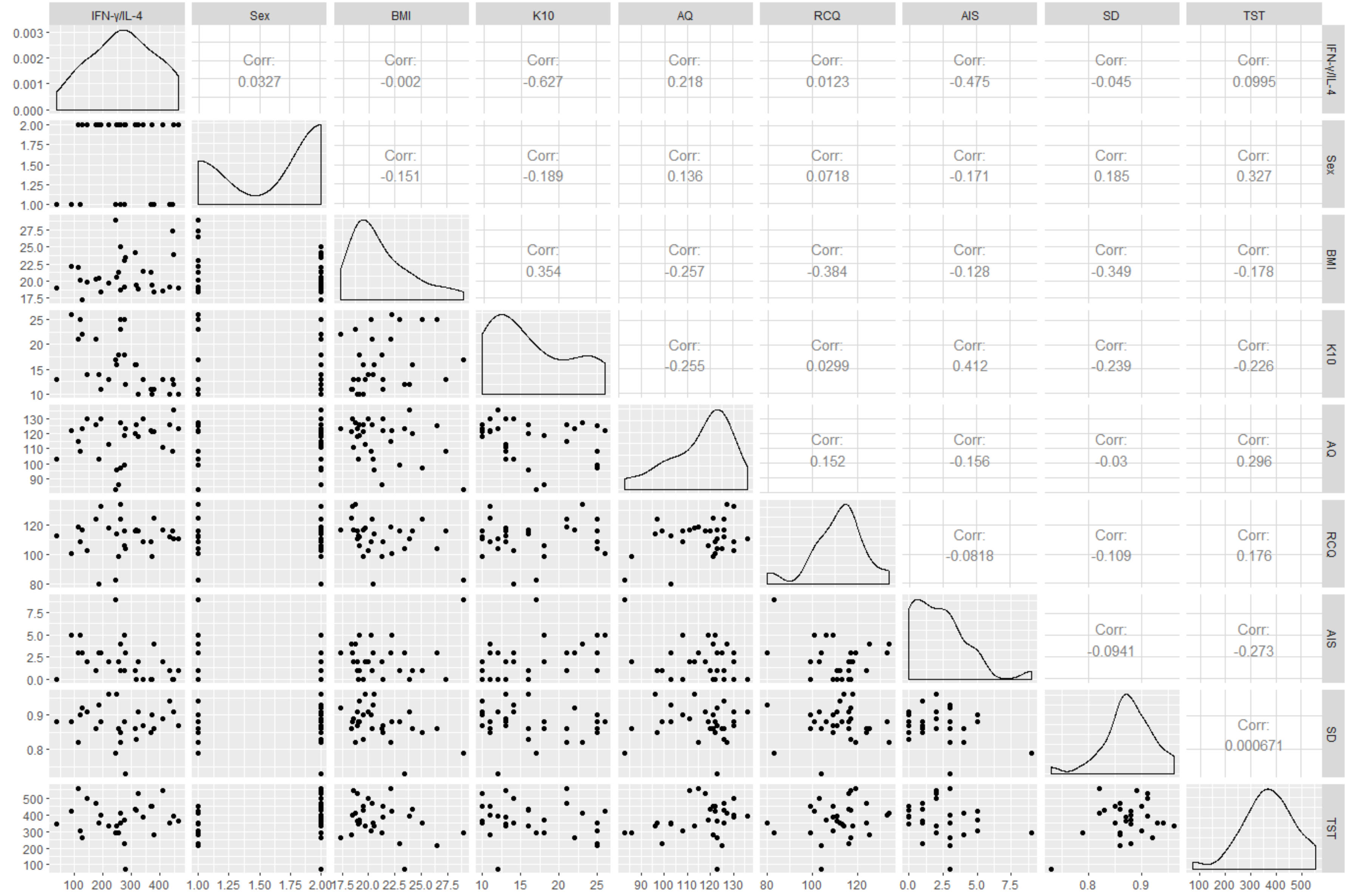

Figure S2: Spearman’s correlation coefficients between IFN-γ/IL-4 and clinical parameters. The distribution of each variable is shown on the diagnal. Below the diagnal, the bivariate scatter plots are displayed. Above the diagnal, the values of the Spearman's correlation coefficients are shown.
